# Integrated analysis of proteome and transcriptome revealed changes in multiple signaling pathways involved in immunity in the northern snakehead (*Channa argus*) during *Nocardia seriolae* infection

**DOI:** 10.3389/fcimb.2024.1482901

**Published:** 2024-12-09

**Authors:** Jian Teng, Yubao Li, Yan Zhao, Yu Zhang, Duanduan Chen, Jianru Liu, Mengyao Cui, Xiangshan Ji

**Affiliations:** ^1^ Phage Research Center, Liaocheng University, Liaocheng, Shandong, China; ^2^ Key Laboratory of Efficient Utilization of Non-grain Feed Resources (Co-construction by Ministry and Province) of Ministry of Agriculture and Rural Affairs, Shandong Agricultural University, Taian, Shandong, China; ^3^ School of Fishery, Zhejiang Ocean University, Zhoushan, Zhejiang, China

**Keywords:** northern snakehead, spleen, proteomics, *Nocardia seriolae*, immune response

## Abstract

The northern snakehead (*Channa argus*) is a valuable aquaculture species across certain Asian countries, contributing significantly to economic prosperity and dietary needs. However, its productivity faces significant challenges, particularly from diseases such as nocardiosis, caused by *Nocardia seriolae*. To date, the majority of research efforts have focused on describing the observed phenomena related to *N. seriolae* infection. However, there remains a notable gap in knowledge concerning the infectivity of *N. seriolae* and the immune response it elicits. To better understand the modulation of the immune responses to *N. seriolae* infection in snakeheads, we investigated the splenic proteome profiles. Specifically, we compared the profiles between uninfected northern snakehead specimens and those infected with *N. seriolae* at 96 h using the label-free data-independent acquisition methodology. A total of 700 differentially expressed proteins (DEPs) were obtained. Of these, 353 proteins exhibited upregulation, whereas 347 proteins displayed downregulation after the infection. The DEPs were mapped to the reference canonical pathways in Kyoto Encyclopedia of Genes and Genomes database, revealing several crucial pathways that were activated following *N. seriolae* infection. Noteworthy, among these were pathways such as ferroptosis, complement and coagulation cascades, chemokine signaling, tuberculosis, natural killer cell-mediated cytotoxicity, and Th17 cell differentiation. Furthermore, protein–protein interaction networks were constructed to elucidate the interplay between immune-related DEPs. These results revealed expression changes in multiple signaling pathways during the initial colonization phase of *N. seriolae.* This discovery offers novel insights into the infection mechanisms and host interaction dynamics associated with *N. seriolae*.

## 1 Introduction

*Nocardia* sp. is a gram-positive, aerobic, filamentous, and non-motile opportunistic pathogen, classified within the phylum *Actinomycetales* (Fatahi-Bafghi, 2018). Nocardiosis, caused by the intracellular *Nocardia* bacteria, is a zoonotic bacterial disease characterized by progressive symptoms such as lethargy, emaciation, and the formation of tissue granulomas (Holland, 2010; Maekawa et al., 2018). Fish nocardiosis is a chronic systemic granulomatous disease, with infected fish displaying symptoms including skin ulceration, muscle necrosis, and organomegaly. In addition, numerous white nodules develop in the gills, heart, liver, kidney, and spleen (Teng et al., 2022; Kim et al., 2018). In aquatic environments, three species of *Nocardia* have been identified in diseased fish and reported as the common pathogenic bacteria of fish nocardiosis. These species are *N. seriolae*, *N. asteroides*, and *N. salmonicida* (Isik et al., 1999; Wang et al., 2014; Tomiyasu, 1982). Notably, outbreaks of fish nocardiosis induced by *N. seriolae* have inflicted substantial economic losses to the aquaculture industry (Isik et al., 1999; Vu-Khac et al., 2016; Wang et al., 2009). *N. seriolae* has emerged as a predominant pathogenic bacterium in aquaculture, contributing to nocardiosis across various freshwater and marine fish species. Notable examples include largemouth bass (*Micropterus salmoides*) (Byadgi et al., 2016), large yellow croaker (*Larimichthys crocea*) (Wang et al., 2005), silver pomfret (*Pampus argenteus*) (Chen et al., 2000), hybrid snakehead (*Channa maculate* ♀ × *Channa argus* ♂) (Chen et al., 2018), yellowtail (*Seriola quinqueradiata*) (Itano et al., 2006) Japanese flounder (*Paralichthys olivaceus*) (Shimahara et al., 2008), and Japanese eel (*Anguilla japonica*) (Kim et al., 2018).

*Channa argus*, commonly known as the northern snakehead, is a widely cultivated and commercially valuable fish species. It is mainly farmed in northern China, as well as in southern and southeastern Asia (Li et al., 2021; Teng et al., 2022). However, *C. argus* is susceptible to many pathogens, particularly *Nocardia seriolae*, which causes outbreaks of nocardiosis disease, which poses a significant threat to the aquaculture of the species (Xu et al., 2017; Chen et al., 2018). Despite significant advances in pathogen research, there remains a glaring absence of effective treatments to restrict the uncontrolled occurrence and rapid spread of nocardiosis in the snakehead aquaculture field (Teng et al., 2022; Hoang et al., 2020). In this context, gaining a deeper understanding of the mechanisms governing the interaction between the host and *N. seriolae* could pave the way for the discovery of new strategies and methods to effectively control nocardiosis. In recent years, the rapid development of proteomics technology, particularly the adoption of mass spectrometry (MS) for quantitative analysis, has played a significant role in studying aquaculture diseases (Schilling et al., 2015; Min et al., 2016). Given that proteins serve as the functional products of gene expression, they are inherently closer to the phenotype than transcripts. Consequently, the resulting proteomic atlas aids in understanding the broader host defense mechanism against pathogen infections. Quantitative proteomics has become a prominent methodology for exploring the host immune response following infection with various pathogens.

In both cultured and natural pond environments, nocardiosis persists as a chronic and systemic disease in fish, characterized by a prolonged survival time from natural infection until death (Teng et al., 2022; Vu-Khac et al., 2016). Our previous findings indicated that *N. seriolae* colonized visceral tissues and initiated the formation of granulomas within snakehead tissues at 3–4 days after the challenge under controlled experimental conditions (data not published). Therefore, sampling at 96 h (4 days) after the challenge may provide a more advantageous time point for analyzing the mechanisms involved in the interaction between the host and *N. seriolae* in snakeheads. Furthermore, the spleen plays a crucial role in fish immune responses and serves as the primary target organ for *N. seriolae* infection and colonization (Xu et al., 2017; Wang et al., 2017). In this study, we used DIA/SWATH (Data-Independent Acquisition/Sequential Window Acquisition of All Theoretical Mass Spectra) technology to characterize the proteome changes in the spleen of northern snakehead 96 h after challenging them with *N. seriolae*. Gene annotation and pathway analysis on differentially expressed proteins (DEPs) were conducted to understand their functions. In addition, the integration of protein and mRNA data in this study offers insights into host immune response events at both transcriptional and posttranscriptional levels.

This study aimed to deepen our understanding of the mechanisms underlying the interaction between the host and *N. seriolae* within the infected spleen. In addition, we aimed to identify specific DEPs with potential to serve as biomarkers in the future, enabling prediction of the outcome of *N. seriolae* infection in aquaculture. These data offer a valuable resource for data mining, facilitating research into the mechanisms of immune response in hosts following *N. seriolae* infection.

## 2 Materials and methods

### 2.1 Fish preparation and bacterial challenge

Northern snakehead specimens (60 ± 5 g) were obtained from a local fish farm in Jining, Shandong, China. Before bacterial infection, the fish were acclimatized to laboratory conditions in two circulating water tanks for 3 weeks. The temperature was maintained at 26 ± 1°C, and the fish were fed commercial feed (Tongwei, China) twice per day. To assure the fish healthy condition, the morphological healthy examination was first performed, and then ten fish were randomly sampled and dissected for examining the healthy condition. The liver and spleen of the fish were examined for pathogenic bacteria using plate counting. The results of the examination confirmed that all fish were healthy and free of pathogenic bacteria.

All experimental procedures received approval from the Shandong Agricultural University Animal Care and Use Committee under approval number SDAUA-2019-016. All animal experiments were carried out according to recommendations in Guide for the Care and Use of Laboratory Animals (eighth edition). All surgical procedures were performed under tricaine methane sulfonate (MS222) (Sigma, Beijing, China), and every possible measure was taken to minimize the suffering experienced by the snakehead specimens.

The *N. seriolae* strain SDAT 0011, previously isolated from diseased northern snakehead, has been maintained in our laboratory (Teng et al., 2022). *N. seriolae* was cultured in fresh brain heart infusion liquid medium and incubated at 28°C for 5 days. After incubation, *N. seriolae* were collected by centrifugation at 3000 rpm and subsequently washed three times with phosphate-buffered saline (PBS). The resulting *N. seriolae* were then homogenized in PBS using a glass homogenizer. The optical density (OD) of the supernatant was measured with an Eppendorf BioPhotometer Plus (Eppendorf, Germany). The method for detecting and calculating the concentration of *N. seriolae* in the supernatant was based on the previous study conducted by Zhang et al. (2024). Prior to the injection assay, the *N. seriolae* suspension was adjusted to a final concentration of 1.0 × 10^7^ CFU mL^−1^ (Teng et al., 2023, 2024).

**Figure 1:**
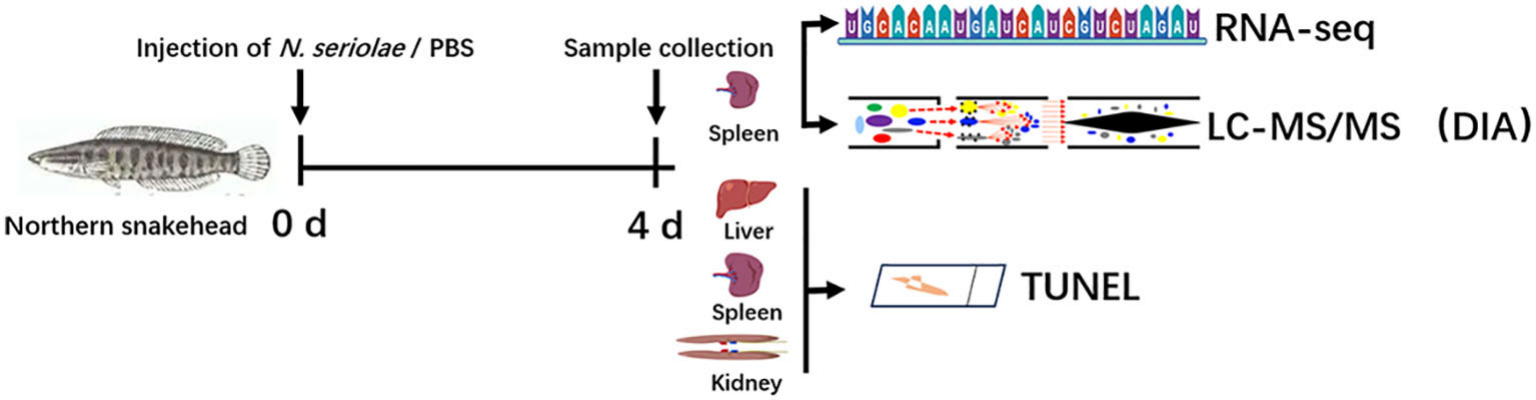
Schematic diagram of the experimental design, indicating the strains used and sampling time points. d: days.

The overall experimental setup is schematically illustrated in **Figure 1**. A total of 20 northern snakeheads were divided randomly into two groups, each containing 10 fish. In the experimental group (N96), each fish was intraperitoneally injected with 100 μL of suspended *N. seriolae*, while in the control group (C96), each fish received an injection of the same volume of PBS. The fish were fed the commercial diet twice daily at 09:00 and 18:00 during the challenge period. At 96 h post infection, the fish were euthanized by anesthetizing them with MS-222 followed by cervical transection. Spleen tissue samples were then randomly collected from three fish in each group and subsequently stored at −80°C until further use. Spleen tissue from three fish in each group was used as three biological replicates. In addition, spleen, kidney, and liver samples from both the N96 and C96 groups were collected and fixed in polyformaldehyde solution for subsequent TdT-mediated dUTP–biotin nick end labeling (TUNEL) assay. No mortalities were observed during the challenge period. The water temperature was maintained at 26 ± 1°C, and water quality parameters (NH_3_ < 0.5, NO_2_ < 0.1, pH 7.6) were routinely monitored using standard test kits (Merck, Germany).

### 2.2 TUNEL assay

To investigate the cellular response in the spleen, liver and kidney following *N. seriolae* infection, a TUNEL assay was used to quantify apoptosis. The TUNEL assay was conducted using the Colorimetric TUNEL Apoptosis Assay Kit (Beyotime, Shanghai, China) following the manufacturer’s protocol. In brief, tissue slices were fixed in acetone for 5 min and then incubated in 20 μg/mL protease K at 37 °C for 10 min. Subsequently, the slices were treated with 3% H_2_O_2_ solution for 10 min at room temperature to deactivate endogenous peroxides. After washing with PBS, the slides were incubated with a mixture solution containing the enzyme terminal deoxynucleotidyl transferase and biotin–dUTP in a humidified atmosphere at 37°C for 60 min. The reaction was terminated by incubating the slides in stop buffer for 10 min at 37°C. After rinsing with PBS, the slices were then incubated with streptavidin–HRP solution for 30 min at room temperature, followed by treatment with DAB for 2–5 min. After light counterstaining with hematoxylin, the slices were dehydrated using ethanol and mounted. Finally, the tissue sections were observed and photographed using a microscope (Nikon, Tokyo, Japan). For the quantitative analysis, four random fields of view were selected from each tissue sample. The number of positively stained cells was counted and expressed as a percentage of the total number of cells within each field of view. The IHC Profiler plug-in for ImageJ was employed to acquire the image data.

### 2.3 Protein extraction, digestion, and high pH reverse phase separation

Total proteins were extracted from the spleen tissues of the C96 and N96 groups using the cold acetone method (Borges et al., 2018). Three biological replicates were evaluated, with three technical replicates per biological replicate. Spleen samples were transferred to a lysis buffer and homogenized on ice for 3 min using an ultrasonic homogenizer. The homogenate was then centrifuged at 4°C for 20 min at 15000 rpm, and the supernatant was subsequently collected. A total of 50 μg of protein extracted from spleen was suspended in 50 μL of solution. The proteins were reduced by the addition of 1 μL of 1 M dithiothreitol and incubated at 55°C for 1 h, followed by alkylation with 5 μL of 20 mM iodoacetamide in the dark at 37°C for 1 h. Samples were precipitated in cold acetone and then resuspended in 50 mM ammonium bicarbonate. Finally, proteins were digested overnight at 37°C with serial grade modified trypsin (Promega, Madison, WI) at a substrate-to-enzyme ratio of 50:1 (w/w).

All peptide samples were dissolved in buffer A, which consisted of 20 mM ammonium formate in water, with pH adjusted to 10.0 using ammonia water, before undergoing separation at high pH. This separation was achieved using an Ultimate 3000 system (Thermo Fisher Science, MA, USA) connected to a 4.6 mm × 250 mm, 5 μm XBridge C18 reverse phase column (Waters Corporation, MA, USA) employing a linear separation method. According to previous studies (Fang et al., 2021), a linear gradient was used, transitioning from 5% B to 45% B over a period of 40 min. Here, B represents a solution containing 80% acetonitrile (ACN) supplemented with 20 mM ammonium formate, with pH adjusted to 10.0 using ammonia water. After each run, the column was re-equilibrated for 15 min. Throughout the separation process, the flow rate was held constant at 1 mL/min, and the column temperature was maintained at 30°C.

### 2.4 DDA qualitative database construction

After desalination and freeze-drying, the peptide segments were redissolved in solvent A, which contained 0.1% formic acid in water, and then subjected to online nanospray liquid chromatography with tandem mass spectrometry (LC-MS/MS). This analysis was conducted on an Orbitrap Fusion Lumos coupled to a Nano ACQUITY UPLC system (Waters Corporation, USA). A total of 4 μL of peptide was loaded onto an Acclaim PepMap C18 analytical column, with a flow rate of 600 nL/min and a column temperature of 40°C. The peptides were separated over a 90-min gradient from 5% B to 32% B, where B represents a solution containing 0.1% formic acid in ACN. An electrospray voltage of 2.1 kV relative to the mass spectrometer inlet was applied.

The Orbitrap Lumos Mass Spectrometer (ThermoFisher Scientific, Bremen, Germany) operated in data-dependent acquisition mode, alternating between MS and MS/MS modes. The parameters were set as follows: (1) MS: scan range (m/z) = 350–1200; resolution = 60,000; AGC target = 400,000; maximum injection time = 50 ms; included charge states = 2–6; filter dynamic exclusion time = 30 s. (2) HCD-MS/MS: resolution = 15,000; AGC target = 50,000; isolation window = 1.2; maximum injection time = 50 ms; collision energy = 32.

### 2.5 Spectral library

The raw data obtained through data-dependent acquisition (DDA) were processed and analyzed using Spectronaut X (Biognosys AG, Switzerland) with default settings. This process generated an initial target list for further analysis. Spectronaut was configured to search the database of *C. argus* (https://www.ncbi.nlm.nih.gov/assembly/GCA_018997905.1/; BioProjects: PRJNA731586), in addition to a contaminant database, assuming trypsin as the digestion enzyme. Carbamidomethyl (C) was designated as the fixed modification, whereas oxidation (M) was specified as the variable modification. Peptides were filtered at a 1% false discovery rate (FDR) and required to have at least one unique occurrence.

### 2.6 DIA data acquisition

Each sample was analyzed by a second acquisition in data independent (DIA) mode. Each sample was dissolved in 30 μL of solvent A, which contained 0.1% formic acid in water. Subsequently, 9 μL of the dissolved sample was mixed with 1 μL of 10RT peptide. These combined samples underwent analysis using online nanospray LC-MS/MS with an Orbitrap Fusion Lumos connected to an EASY-nLC 1200 system (Thermo Fisher Scientific). The peptides (3 μL) were loaded onto the analytical column and separated using a gradient from 5% B to 35% B over a 120-minute period. Here, B denotes a solution of 0.1% formic acid in ACN. The column flow rate was set at 200 nL/min, and the electrospray voltage was adjusted to 2 kV. The mass spectrometer operated in DIA mode, automatically alternating between MS and MS/MS modes. Full-scan MS spectra (m/z 350-1200) and high energy collisional dissociation (HCD) MS/MS were acquired with a mass resolution of 12000 and 30000, respectively. The automatic gain control (AGC) target value and maximum injection time for MS were set to 1e6 and 50 ms, respectively. The collision energy and stepped collision energy for HCD-MS/MS were established at 32 and 5%, respectively. The collision energy and stepped collision energy for HCD-MS/MS were established at 32% and 5%, respectively. DIA was performed using a variable isolation window, with each window overlapping by 1 m/z, resulting in a total of 60 windows.

### 2.7 Data analysis

The raw data obtained through data-independent acquisition (DIA) were processed and analyzed using Spectronaut X (Biognosys AG) with BGS Factory Settings. Retention time prediction was configured as dynamic iRT. Data extraction was performed in Spectronaut X with extensive mass calibration. Additional data searches were carried out using Spectronaut Pulsar X (Biognosys, Switzerland). A *q*-value (FDR) cutoff of 1% was applied to both precursor and protein levels. Decoy generation was configured to mutated, a parameter akin to scrambled, but instead applies a random number of AA position swamps (minimum = 2, maximum = length/2). All selected precursors that passed the filters were used for quantification. The major group quantities were determined by calculating the averages from the top three filtered precursors that passed the 1% *q*-value cutoff. Subsequently, the normalized expression level of each protein was determined as the median of all unique peptides annotated to that protein. A Student’s *t*-test was then conducted, and proteins showing a fold change >1.5 or <0.67 in Cp-vs-Np, with an adjusted significance level of *P* < 0.05, were identified as differentially expressed. Mass spectrometry data from this study have been deposited in the Proteome Xchange Consortium database under the project ID PXD041478.

### 2.8 Bioinformatics analysis

Protein identification and quantification were performed following a previously published protocol (Pan et al., 2015). Proteins were annotated utilizing the NCBI non-redundant (NR) and Swiss-Prot databases. The functional descriptions of identified protein domains were annotated by InterProScan based on protein sequence alignment methods and the InterPro domain database. Proteins were classified using the Gene Ontology (GO), Kyoto Encyclopedia of Genes and Genomes (KEGG), and eukaryotic Ortholog Groups (KOG) databases. A significance threshold of *P*-value ≤ 0.05 was applied to determine the statistically significant enrichments of GO and KEGG pathways. Information regarding protein–protein interactions for the DEPs was acquired using the STRING online software platform (https://string-db.org/). The resulting network was visualized using Cytoscape software (National Resource for Network Biology, United States) (Shannon et al., 2003).

### 2.9 Integration analysis of proteome and transcriptome

The OmicShare tools were used to perform an integrated study of proteome and transcriptome. The log2 transformation of the average fold changes was obtained for the transcriptome and proteome data. Subsequently, the transcriptomic and proteomic datasets were filtered to create a combined dataset for further analysis. The following parameters were utilized: P ≤ 0.05 and fold change > 1.5 for both mRNA and protein. The resulting combined dataset comprises the union of DEGs and DEPs that meet the specified filtering criteria. Consequently, this dataset includes overlapping genes, as well as unique DEGs and unique DEPs. Based on data in the combined dataset, a nine-quadrant map was constructed. Additionally, GO and KEGG analyses were conducted on the genes within the combined dataset.

### 2.10 Quantitative real-time PCR

Total RNA was extracted from Cp and Np samples using TRIzol reagent (Invitrogen, USA), following the protocol described by Teng et al. (2022). cDNA was synthesized using the HiScript III 1st Strand cDNA Synthesis Kit (Vazyme, Nanjing, China) and used as a template for quantitative real-time polymerase chain reaction (qRT-PCR) with the SYBR^®^ Green Premix *Pro Taq* HS qPCR Kit (Accurate Biotechnology (Hunan) Co., Ltd., ChangSha, China) following the manufacturer’s instructions. qRT-PCR assays were performed on a Roche LightCycler^®^96 instrument. Specific primers were designed using Primer-Blast, based on previous transcriptome data (NCBI Accession Number: SRP302800). The primer sequences are available in **Supplementary Table S1** of **Additional File 1**. The internal control gene used was *β-actin*. Each sample was tested in triplicate, and the fold change of target genes was determined using the 2^−ΔΔCt^ method. Statistical analysis was conducted using one-way Analysis of Variance (ANOVA) followed by *post hoc* Duncan’s multiple range tests in SPSS 22.0 to compare the two groups.

### 2.11 Western blotting validation

Following the previous DIA proteomics results, three key proteins were selected for further validation through additional Western blotting analysis. Total proteins were extracted using a Protein Extraction Kit (Solarbio, Beijing, China) following the manufacturer’s instructions. The protocol for the preparation of antiserum was based on previous studies. In brief, New Zealand rabbits were injected subcutaneously with recombinant protein (1 mg per rabbit) emulsified with Freund’s complete adjuvant. On day 15 following the initial injection, the rabbits received an additional 0.5 mg of recombinant protein emulsified in Freund’s incomplete adjuvant (Sigma). The polyclonal antiserum was collected on day 10 after injection, and the antibodies were purified using Protein A Agarose (GE Healthcare, Shanghai, China). The specificity of antibodies against LECT2, NCCRP1, and GAPDH has been confirmed in previous studies (Teng et al., 2024, 2023). The MPEG1 protein was analyzed for antigenic fragments using the NovoFocus tool. Following the preparation of the antibody, a titer test was performed using the ELISA method, revealing an antibody titer of 1:25600. Additionally, the Western Blot results confirmed the specificity of the prepared anti-MPEG1 antibodies (**Supplementary Figure S1**).

Proteins were separated by 10% SDS-PAGE and transferred onto polyvinylidene fluoride membranes (Millipore, Temecula, CA, USA). After a 2-hour blocking step with blocking buffer, the membranes were incubated with antiserum against MPEG1 (macrophage-expressed gene 1, 1:200, diluted in blocking buffer), NCCRP1 (nonspecific cytotoxic cell receptor 1, diluted at 1:500), LECT2 (leukocyte cell-derived chemotaxin 2, diluted at 1:200), or GAPDH (Beyotime, 1:500) for 2 h at room temperature. The antibodies were raised by inducing the expression of recombinant proteins and immunizing New Zealand rabbits to obtain antiserum. Finally, Horseradish peroxidase (HRP)-conjugated goat anti-rabbit IgG (diluted at 1/1000 in TBS) was added and incubated for 1 hour before visualizing the results on a Fusion-SL7 system (Vilber Lourmat, Collégien, France).

## 3 Results

### 3.1 Apoptosis in tissues after *N. seriolae* infection

**Figure 2:**
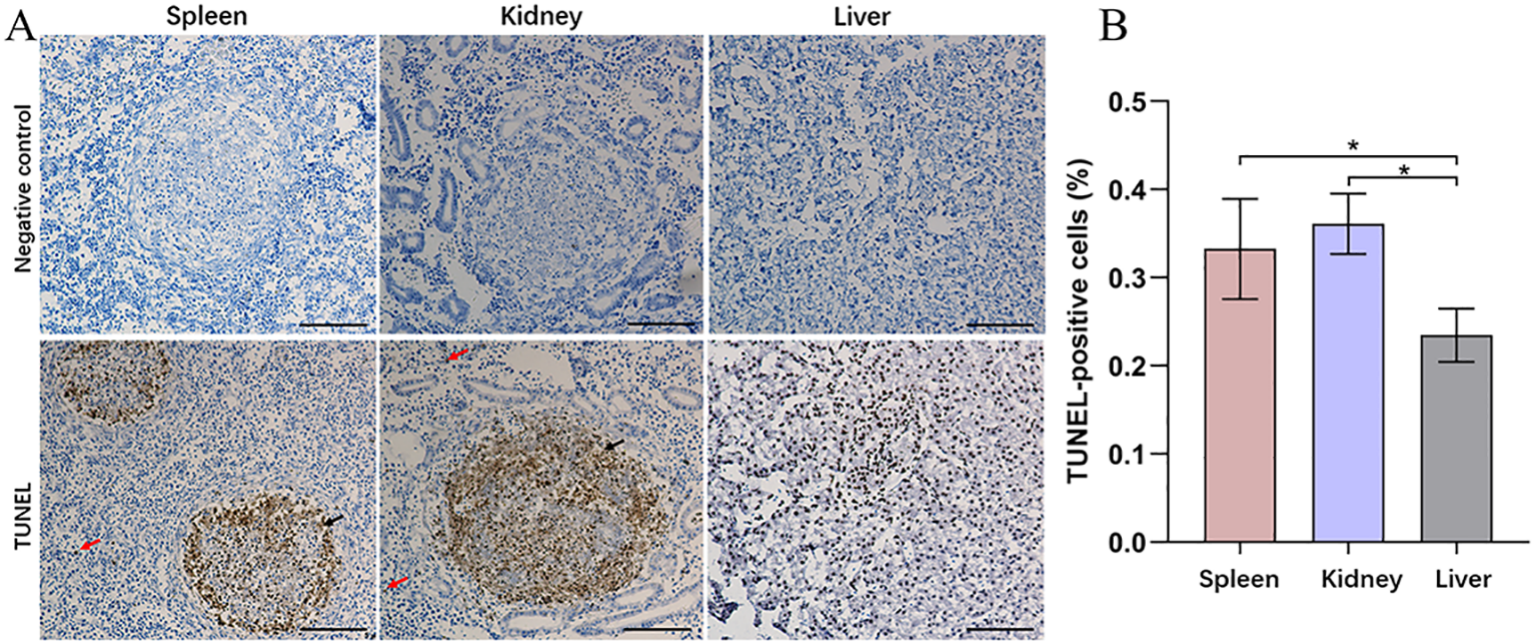
Apoptosis was investigated in the spleen, kidney, and liver of northern snakehead fish after *N. seriolae* infection using TUNEL staining. **(A)** Representative images of TUNEL; **(B)** quantitative analysis of TUNEL-positive cells. The presence of a positive signal was visualized by a brownish color. The positive signal corresponds to the brownish color. Negative control: without treatment. Red arrows indicate apoptotic cells located outside the granuloma, while black arrows denote apoptotic cells within the granuloma. Nuclei were stained with hematoxylin. *P < 0.01. Scale bar = 5 µm.

Prior to investigating the immune response events in snakeheads after *N. seriolae* infection, we considered cell survival in the visceral tissues. This consideration was prompted by observed symptoms in the fish, such as skin hemorrhage, intra-ocular hemorrhage, and abdominal swelling at 3–4 days post infection (dpi). At 9 dpi, numerous granulomas were observed in the spleen and kidneys of infected snakeheads. TUNEL staining revealed no positive signals in the negative control group (**Figure 2A**). Interestingly, a substantial number of TUNEL-positive cells were identified within the granulomas in both the spleen and kidney post *N. seriolae* infection, indicating widespread apoptosis within the granuloma. Conversely, only a few positive cells were found in tissues outside the granulomas. Liver sections did note display any granulomas, yet numerous TUNEL-positive cells were scattered throughout the liver, as revealed by TUNEL staining. Quantitative analysis demonstrated a significantly higher number of apoptotic cells in the spleen and kidney compared to those in the liver (**Figure 2B**).

### 3.2 Clustering heatmap analysis of DEGs in the transcriptome, and proteome analysis

**Figure 3:**
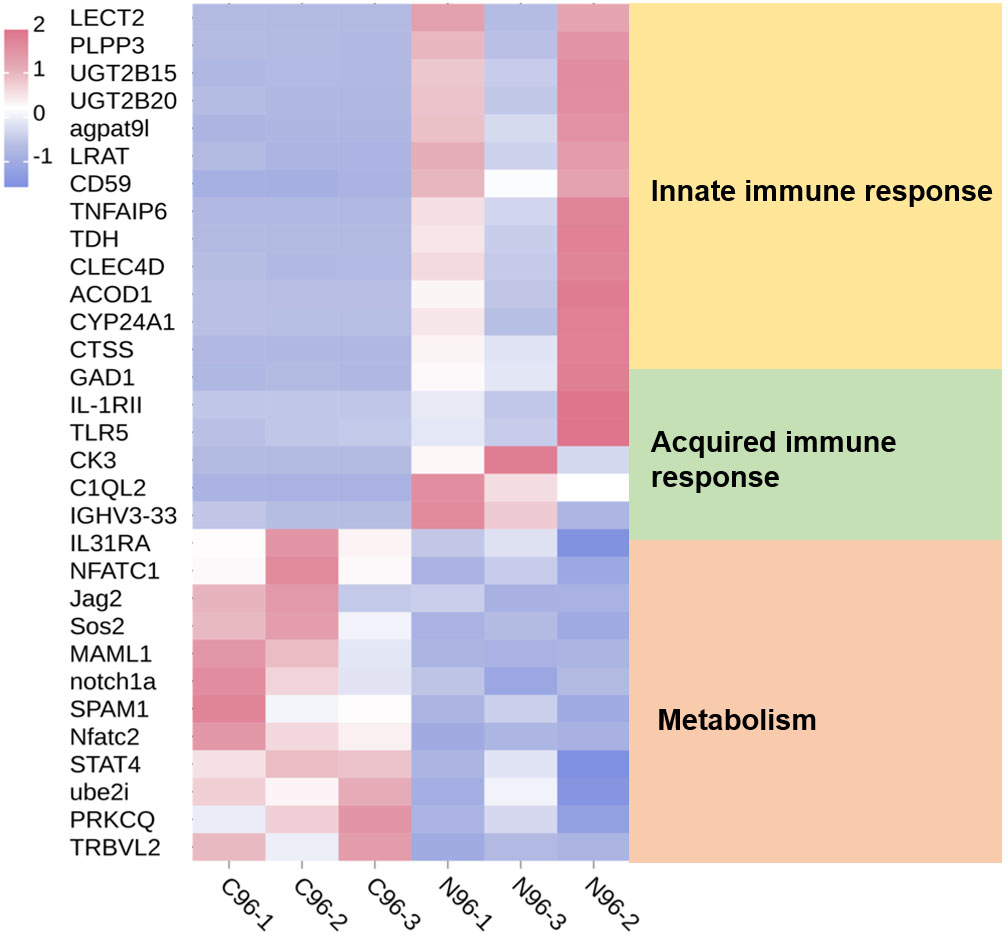
Heatmaps analysis of DEGs with different known functions in spleen tissues. The intensity of the color from blue to red indicates the magnitude of differential expression; red and blue indicate up- and down-regulation, respectively. The mapping data were derived from previously published transcriptome data. Transcriptome analysis of the spleen of snakeheads was performed at 96 days post-infection (dpi). N96: 96 hours after *N. seriolae* infection. C96: Control group.

Based on previous transcriptomic data from the spleen (Teng et al., 2022), the annotated DEGs are presented as a clustered heatmap (**Figure 3**). The DEGs that exhibited significant changes following infection are primarily associated with innate immunity, acquired immunity, and metabolism. Notably, a substantial number of immune-related DEGs were significantly upregulated in the N96 group. Consequently, we conducted a proteomic analysis to further investigate the immune response events of host cells in response to *N. seriolae* infection.

The library used for DIA analysis of label-free quantitative proteomics in northern snakehead spleen samples has been constructed. Following strict thresholds (FDR < 0.01), a total of 70,671 precursors, 63,305 peptides, 7,688 protein groups, and 7,825 proteins were identified (**Supplementary Figure S2A**). Analysis showed that more than half of the peptides were composed of five or fewer peptides, and over 76.24% of proteins consisted 10 or fewer peptides. Only 23.75% of the proteins were composed of 11 or more polypeptides (**Supplementary Figure S2B**). Before mass spectrometric analysis, the raw DIA data underwent processing and analysis using Spectronaut X (Biognosys AG, Switzerland). This involved configuring spectral library parameters to ensure a high confidence level for the DIA conducted in this study. The results indicated that fragment information was complete for all ions following extensive mass calibration and data filtering. Moreover, the signal intensity demonstrated consistent response levels across all samples (**Supplementary Figure S2C**). Among the identified and quantified proteins, 700 DEPs were detected (P-value < 0.05). Of these proteins, 353 were upregulated, whereas 347 were downregulated in Cp compared with Np (**Supplementary Figure S3A**; **Additional File 2**). Furthermore, a heatmap was generated to visualize the expression abundance of DEPs across the six proteomic analysis samples (**Supplementary Figure S3B**).

### 3.3 GO classification, functional enrichment, and protein domain analysis of DEPs

**Figure 4:**
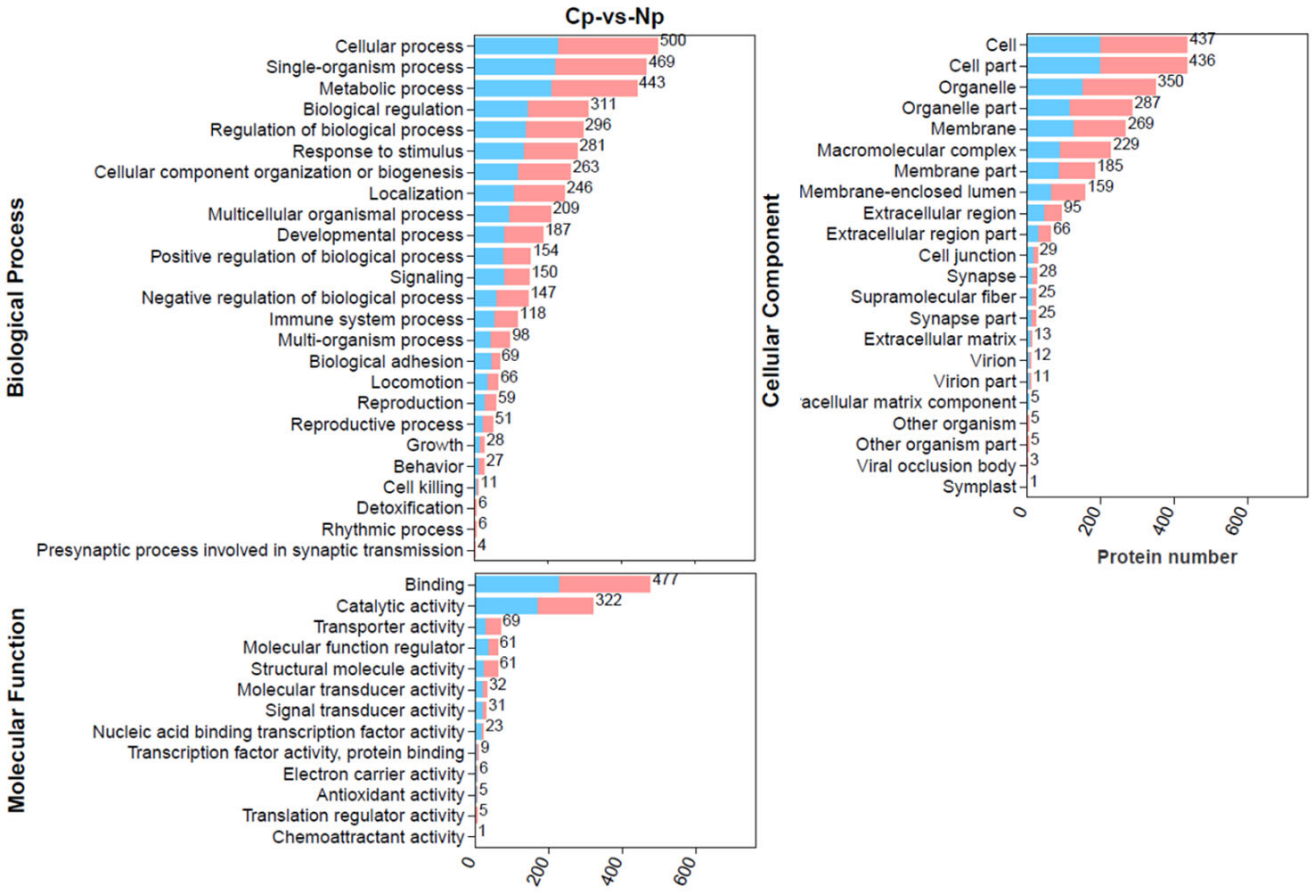
Gene Ontology (GO) annotation terms of differentially expressed proteins (DEPs) at GO level 2. The pink bar and blue bar represent up- and downregulated proteins, respectively.

To explore the detailed functional classification of these DEPs, we conducted a comprehensive analysis by mapping all DEPs to the GO database and performing GO enrichment analysis (**Figure 4**). In total, 700 DEPs were categorized into 25 biological processes, 22 cellular components and 13 molecular functions. In the biological process category, the majority of DEPs were involved in cellular processes, single-organism processes, and metabolic processes. In the molecular function category, the top four enriched terms were binding, catalytic activity, transporter activity, and molecular function regulator. In the cellular component category, the top four enriched terms were cell, cell part, organelle, and organelle part.

**Figure 5:**
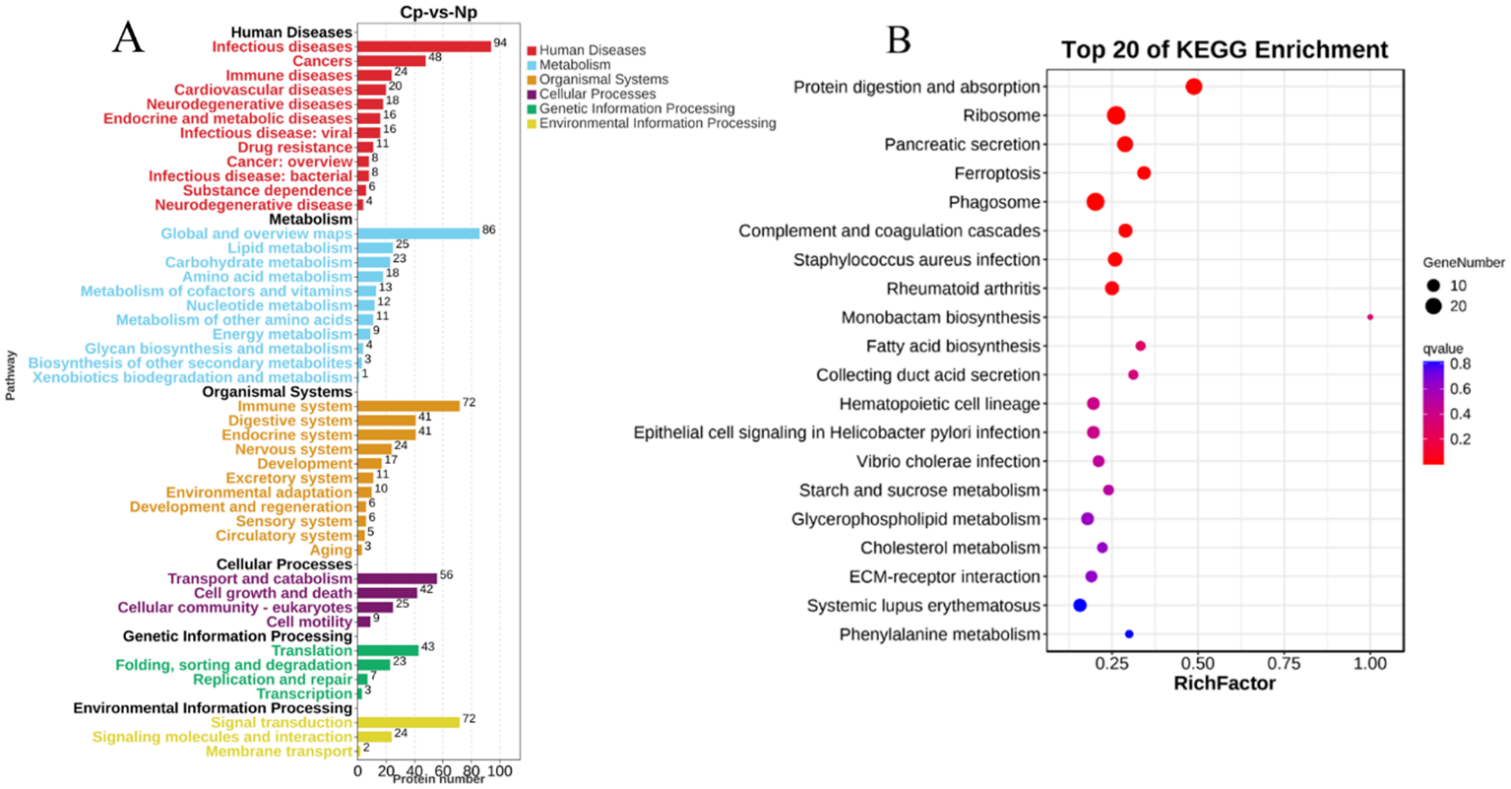
Pathway enrichment of DEPs via KEGG analysis. **(A)** KEGG pathways analysis of identified proteins. The Y-axis indicates the pathway classification (2nd level) and the X-axis indicates the number of differentially expressed proteins (DEPs). **(B)** Scatterplot of the top 20 enriched KEGG pathways. The X-axis represents the rich factor of the pathway, while the Y-axis shows various KEGG pathways.

A comparison with the KEGG database revealed that the 700 DEPs were assigned to 308 pathways. All the annotated pathways were grouped into six major categories. Within these categories, the dominant KEGG subcategories were infectious diseases, global and overview maps, immune system, transport and catabolism, translation, and signal transduction in the Cp versus Np comparison groups (**Figure 5A**). In the immune system subcategory, 72 immune system-related pathways were differentially expressed in the Cp versus Np comparison groups (**Figure 5A**). The represented immune-related pathways included complement and coagulation cascades, chemokine signaling pathway, Th17 cell differentiation, natural killer cell-mediated cytotoxicity, C-type lectin receptor signaling pathway, and B cell receptor signaling pathway Among them, the complement and coagulation cascades (13 DEPs) and chemokine signaling pathway (13 DEPs) in the immune system subcategory had the largest number of DEPs (**Table 1**, *p* < 0.01). In addition, **Figure 5B** illustrates the top 20 of pathways with the highest abundance of DEPs related to *N. seriolae* infection. These pathways include protein digestion and absorption, ribosome, pancreatic secretion, ferroptosis, phagosome, and complement and coagulation cascades.

**Table 1:** Immune-related DEP expression in northern snakehead after *N. seriolae* infection.

ID	Category/Gene name	Description	Fold change
Ferroptosis			
Carg_Chr02G011120.1	FTH1	Ferritin heavy chain	3.27
Carg_Chr23G005500.1	FTL	Ferritin light chain	3.27
Carg_Chr17G005140.1	SAT2	Diamine acetyltransferase 2	1.64
Carg_Chr08G005380.1	ACSL1	Long-chain-fatty-acid--CoA ligase 1	-1.99
Carg_Chr16G002220.1	ACSL3	Long-chain-fatty-acid--CoA ligase 3	1.58
Carg_Chr17G002570.1	Slc3a2	4F2 cell-surface antigen heavy chain	2.07
Carg_Chr11G008510.1	CP	Ceruloplasmin	1.73
Carg_Chr16G005010.1	TFR1	Transferrin receptor protein 1	2.37
Carg_Chr20G007920.1	VDAC2	voltage-dependent anion-selective channel protein 2	-1.50
Complement and coagulation cascades			
Carg_Chr04G011370.1	–	uncharacterized protein LOC108877178	1.69
Carg_Chr09G007340.1	MASP2	Mannose associated serine protease 2	1.76
Carg_Chr03G008280.1	CFD	Complement factor D	-1.70
Carg_Chr19G000810.1	Bf	Complement factor B	2.18
Carg_Chr12G002920.1	VWF	von Willebrand factor	-1.54
Carg_Chr11G009450.1	Serpinc1	Antithrombin-III	-1.6
Carg_Chr10G001120.1	SERPINF2	alpha-2-antiplasmin	2.40
Carg_Chr07G010500.1	C1qb	Complement C1q subcomponent subunit B	3.17
Carg_Chr07G010510.1	C1qc	Complement C1q subcomponent subunit C	2.04
Carg_Chr12G010380.1	C4BPA	CUB and sushi domain-containing protein 1 CUB and sushi multiple domains protein 1 Precursor	-1.94
Carg_Chr11G009040.1	CFH	Complement factor H H factor 1 Precursor	1.81
Carg_Chr13G000010.1	ITGB2	Integrin beta-2 Cell surface adhesion glycoprotein	-1.52
Chemokine signaling pathway			
Carg_Chr02G008230.1	STAT3	Signal transducer and activator of transcription 3	1.59
Carg_Chr08G002340.1	RAC2	Ras-related C3 botulinum toxin substrate 2	-1.54
Carg_Chr08G009550.1	GNG12	Guanine nucleotide-binding protein G(I)/G(S)/G(O) subunit gamma-12	-1.6
Carg_Chr12G005050.1	PIK3CD	Phosphatidylinositol 4,5-bisphosphate 3-kinase catalytic subunit delta	-2.01
Carg_Chr12G009450.1	WASP	wiskott-Aldrich syndrome protein	-1.6
Carg_Chr14G004540.1	DOCK2	Dedicator of cytokinesis protein 2	-1.53
Carg_Chr15G008480.1	JAK2	Tyrosine-protein kinase JAK2	-1.98
Carg_Chr15G010320.1	CXCL13	C-X-C motif chemokine 13	-4.19
Carg_Chr16G006130.1	LYN	Tyrosine-protein kinase Lyn	2.26
Carg_Chr17G000130.1	ARRB2	arrestin, beta 2b	1.67
Carg_Chr17G001120.1	CCL4	C-C motif chemokine 4 -like protein Macrophage inflammatory protein 1	-2.67
Carg_Chr18G001450.1	NCF1	Neutrophil cytosolic factor 1	-1.91
Carg_Chr24G000850.1	rap1b	Ras-related protein Rap-1b	-1.81
Tuberculosis			
Carg_Chr09G011070.1	CTSS	Cathepsin S	3.51
Carg_Chr12G010950.1	ATP6AP1	V-type proton ATPase subunit S1	1.71
Carg_Chr12G010380.1	C4BPA	CUB and sushi domain-containing protein 1 CUB and sushi multiple domains protein 1 Precursor	-1.94
Carg_Chr23G007790.1	mapk8	mitogen-activated protein kinase 8 isoform X4	1.58
Carg_Chr15G008480.1	Jak2	Tyrosine-protein kinase JAK2	-1.98
Carg_Chr15G009370.1	Camk2b	calcium/calmodulin-dependent protein kinase type II subunit beta	-1.62
Carg_Chr07G001260.1	BPI	Bactericidal permeability-increasing protein	1.68
Carg_Chr01G011880.1	PPP3R1	Calcineurin subunit B type 1, partial	-2.33
Carg_Chr13G000010.1	ITGB2	Integrin beta-2 Cell surface adhesion glycoprotein	-1.52
Carg_Chr10G002650.1	FcERI	High affinity immunoglobulin epsilon receptor subunit alpha Fc-epsilon RI-alpha	2.45
Carg_Chr16G007610.1	MRC1	Macrophage mannose receptor 1	2.69
Carg_Chr17G009800.1	H2-Eb1	H-2 class II histocompatibility antigen, A-K beta chain	-2.06
Carg_Chr02G004350.1	–	Ig mu chain C region	-3.31
Carg_Chr15G008560.1	MALT1	Mucosa-associated lymphoid tissue lymphoma translocation protein 1	-1.84
Carg_Chr12G009920.1	CEBPβ	CCAAT/enhancer-binding protein beta	3.47
Carg_Chr01G000260.1	CLE4E	C-type lectin domain family 4 member E	19.56
Carg_Chr15G011520.1	Card9	Caspase recruitment domain-containing protein 9	1.62
Carg_Chr02G009290.1	CORO1A	Coronin-1A	-1.63
Natural killer cell mediated cytotoxicity			
Carg_Chr12G005050.1	PIK3CD	Phosphatidylinositol 4,5-bisphosphate 3-kinase catalytic subunit delta	-2.01
Carg_Chr08G002340.1	RAC2	Ras-related C3 botulinum toxin substrate 2	-1.54
Carg_Chr04G011400.1	CD247	T-cell surface glycoprotein CD3 zeta chain	-1.55
Carg_Chr04G000480.1	PLCG2	1-phosphatidylinositol 4,5-bisphosphate phosphodiesterase gamma-2	-1.63
Carg_Chr01G011880.1	PPP3R1	Calcineurin subunit B type 1, partial	-2.33
Carg_Chr13G000010.1	ITGB2	Integrin beta-2 Cell surface adhesion glycoprotein	-1.52
Carg_Chr02G004350.1	–	Ig mu chain C region	-3.31
Carg_Chr11G007700.1	Zap70	Tyrosine-protein kinase ZAP-70	-1.70
Carg_Chr11G007700.1	Prf1	Perforin-1	-2.35
Carg_Chr12G010870.1	Nfatc2	Nuclear factor of activated T-cells, cytoplasmic 2	-1.91
Th17 cell differentiation			
Carg_Chr17G009800.1	H2-Ab1	H-2 class II histocompatibility antigen, A-K beta chain	-2.06
Carg_Chr03G007710.1	TRBC1	T-cell receptor beta-1 chain C region	-1.83
Carg_Chr23G007790.1	mapk8	mitogen-activated protein kinase 8	1.58
Carg_Chr15G008480.1	Jak2	Tyrosine-protein kinase JAK2	-1.98
Carg_Chr02G008230.1	Stat3	Signal transducer and activator of transcription 3	1.59
Carg_Chr01G011880.1	PPP3R1	Calcineurin subunit B type 1, partial	-2.33
Carg_Chr04G011400.1	CD247	T-cell surface glycoprotein CD3 zeta chain	-1.55
Carg_Chr11G007700.1	Zap70	Tyrosine-protein kinase ZAP-70	-1.70
Carg_Chr16G001080.1	IRF4	Interferon regulatory factor-4	-1.92
Carg_Chr12G010870.1	NFATC2	Nuclear factor of activated T-cells, cytoplasmic 2	-1.91

**Figure 6:**
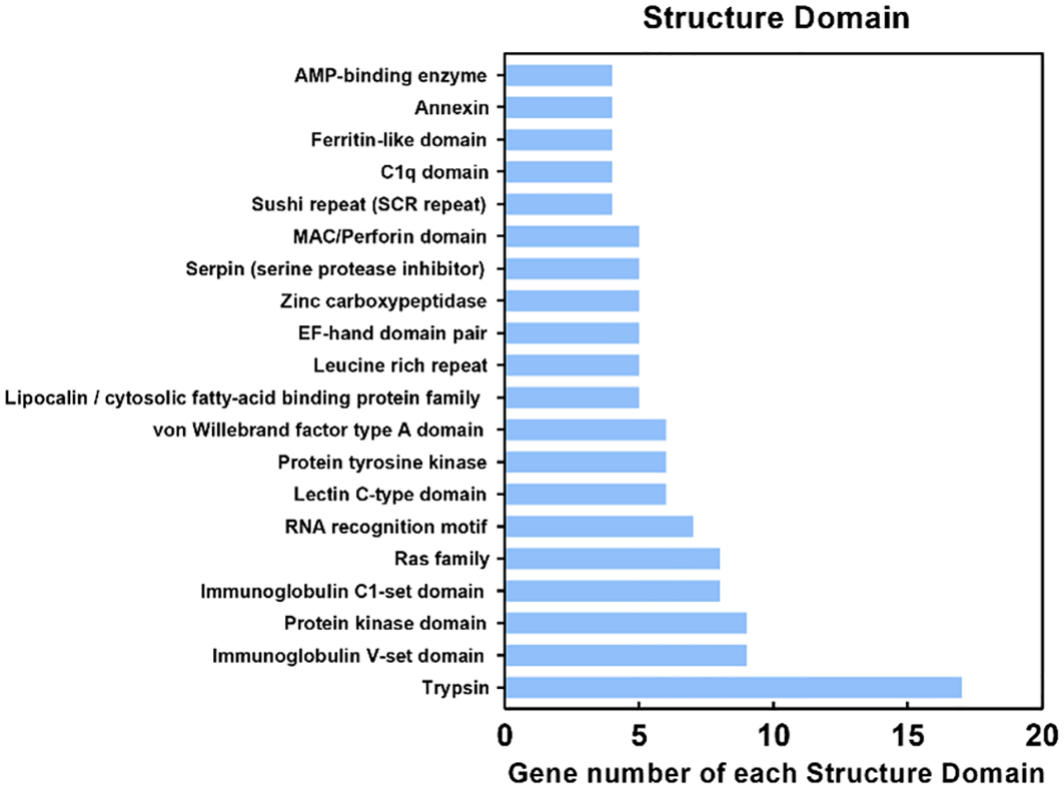
Protein domain (Pfam) analysis of identified proteins. The Y-axis indicates the domain and the X-axis indicates the number of differentially expressed proteins (DEPs). The top 20 protein domains are shown.

The DEPs were submitted to the Pfam database for analysis using Hidden Markov Models to predict the protein domains they possess. The results showed that 700 proteins contained various structural domains (**Figure 6**), including trypsin (17 DEPs), immunoglobulin V-set domain (9 DEPs), protein kinase domain (9 DEPs), immunoglobulin C1-set domain (8 DEPs), ras family (8 DEPs), RNA recognition motif (7 DEPs), lectin C-type domain (6 DEPs), and ferritin-like domain (4 DEPs). Notably, many of these structural domains are involved in antimicrobial immunity.

### 3.4 Construction of protein–protein interaction networks and identification of key immune-related DEPs

**Figure 7:**
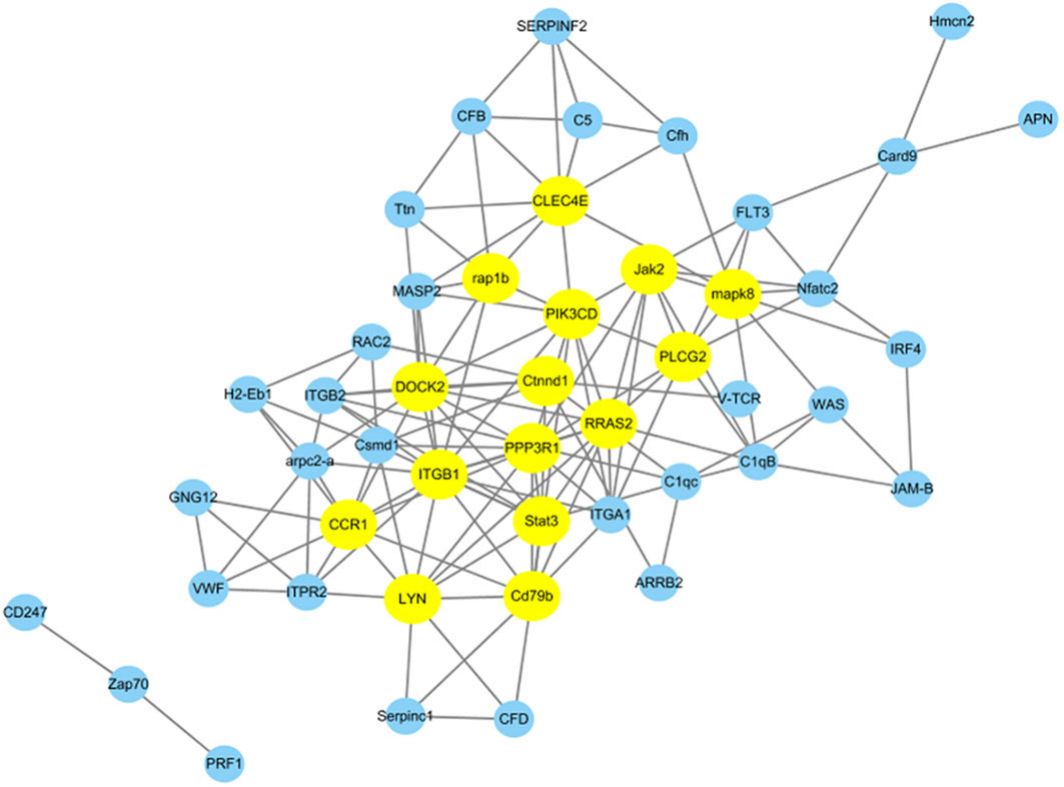
Protein-protein interaction networks were analyzed among differentially abundant proteins. A total of 47 DEPs from 21 immune system-related pathways in the Cp96-vs-Np96 group with *P*-value <0.05 and Pearson >∣0.90∣are chosen for network display. The yellow nodes in the network represent the top 15 immune-related proteins based on Pearson correlation.

We conducted additional analysis to explore gene co-expression networks to find the hub and key genes crucial for immune responses. Utilizing 47 DEPs from 21 immune system-related pathways, we constructed gene co-expression networks to uncover their patterns and interactions within the immune system. In **Figure 7**, the top 15 key genes identified based on Pearson correlation are highlighted with specific colors. Among these key genes, *Clec4e*, *Lyn*, *Stat3*, *mapk8*, and *Ctnnd1* were found to be upregulated at 96 h after *N. seriolae* infection. Furthermore, *Jak2*, *Rap1b*, *Plcg2*, *Cd79b*, *CCR1*, and *PPP3R1* were downregulated at the same time point following *N. seriolae* infection. These genes play roles in immune regulation and demonstrate a high level of connectivity in the biological network. Further research on these genes is necessary to gain a deeper understanding of their specific contributions to the immune response against *N. seriolae* infection.

### 3.5 Integration analysis of proteome and transcriptome

**Figure 8:**
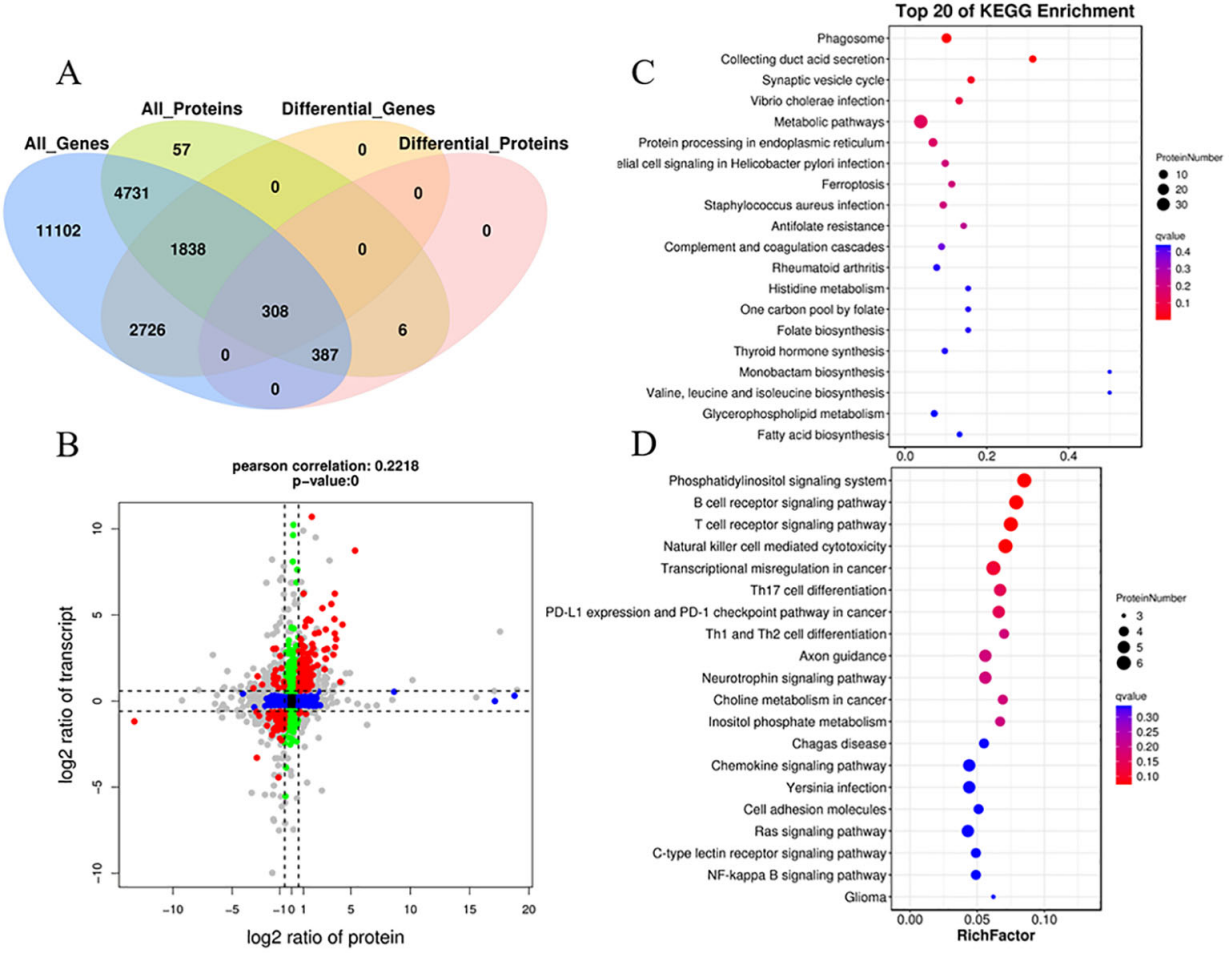
Correlation analysis of transcriptomic and proteomic differences in snakeheads infected with *N. seriolae* compared to uninfected controls. **(A)** A Venn diagram illustrating the shared and unique genes among the different sample groups. **(B)** Correlation analysis of transcriptome and proteome differences between Cp and Np groups. (Both protein and mRNA fold change >1.5). Red dots (group 1, 3, 7, and 9) represent significant changes of expression in both mRNA and protein; Green dots (group 2 and 8) show significant changes in mRNA expression levels only; Blue dots (group 4 and 6) denote significant changes of expression in protein levels only; Black dots (group 5) display no significant change of expression in either mRNA or proteins; The gray dots denote that the fold change reached the threshold, while the P-value did not. **(C)** KEGG pathway analysis of group 3. **(D)** KEGG pathway analysis of group 7. The Y-axis represents pathway classification (2nd level) and the X-axis indicates the number of genes.

Integrating transcriptomic and proteomic analyses can provide valuable insights for identifying key genes of interest. The transcriptome data used for the integrated analysis were obtained from previous studies (Teng et al., 2022). A total of 7,264 co-expressed genes and proteins were identified (**Figure 8A**). Moreover, a total of 308 co-expressed DEGs/DEPs (with fold change >1.5 or <0.67) were found in the two comparison groups (**Figures 8A, B**; **Additional file 3**). The distribution of these co-expressed DEGs/DEPs, shown as red dots, is notably concentrated in quadrants 3 and 7, surpassing the count in quadrants 1 and 9. This indicates a prevalent positive correlation between protein abundances and mRNA enrichment for most co-expressed DEGs/DEPs (286 genes, 92.9%) (**Figure 8B**). In quadrants 3 and 7, the consistent change in mRNA and protein levels implies that the expression of most proteins is regulated at the transcriptional level following *N. seriolae* infection. The expression patterns of the nine selected DEGs identified by qRT-PCR closely matched the protein expression profiles analyzed through sequencing (**Supplementary Figure S4**). In quadrant 3, the identified genes represent upregulated proteins predominantly enriched in the phagosome, synaptic vesicle cycle, *Vibrio cholerae* infection, protein processing in endoplasmic reticulum, epithelial cell signaling in *Helicobacter pylori* infection, and the ferroptosis pathway (**Figure 8C**; **Supplementary Figure S5**). In quadrant 7, the identified genes represent downregulated proteins mainly enriched in the immune system subcategory. This includes pathways such as the B cell receptor signaling pathway, T cell receptor signaling pathway, natural killer cell-mediated cytotoxicity, and Th17 cell differentiation (**Figure 8D**). These annotations offer valuable resources for further exploring the immune response in the host.

### 3.6 Validation the expression of significant proteins

**Figure 9:**
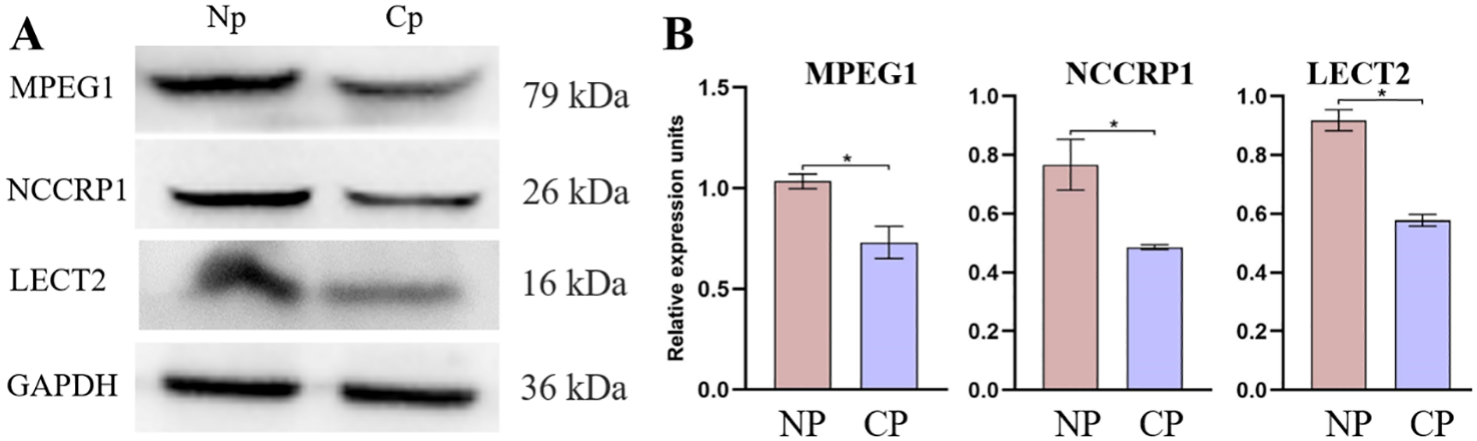
Protein expression abundance of LECT2, NCCRP1, and MPEG1 was validated in the CP and Np groups using western blot analysis, with GAPDH as the loading control. **(A)** Representative western blot analysis and quantitative results **(B)**. MPEG1: macrophage-expressed gene 1; *NCCRP1*: nonspecific cytotoxic cell receptor 1; *LECT2:* leukocyte cell-derived chemotaxin 2. *P < 0.01.

To further validate the proteomics data, western blotting was conducted for MPEG1, NCCRP1, and LECT2 proteins (**Figure 9A**). The validation results revealed a marked increase in the expression abundance of MPEG1, NCCRP1 and LECT2 proteins in the NP group compared to the CP group (**Figure 9B**). Specifically, the proteomic data indicated that the expression levels of MPEG1, NCCRP1, and LECT2 proteins were up-regulated by 1.02, 1.38, and 3.26-fold, respectively, in the NP group relative to the CP group. These validation results were generally consistent with the trends observed in the proteomics data.

## 4 Discussion

In this study, we used DIA/SWATH technology for the first time to investigate proteomic responses to *N. seriolae* infection in the spleen of northern snakeheads. This approach aimed to elucidate host–pathogen interactions and downstream immune events triggered by infection. Moreover, we highlight that quantitative proteomics offers insights that are not attainable through transcriptomic data alone. By examining the relationship between proteomic and transcriptomic data, this study offers evidence of regulatory events at both transcriptional and posttranscriptional levels following spleen infection with *N. seriolae*. The research presents an accurate measurement and comprehensive interpretation of protein expression in the spleen of infected northern snakeheads during the initial colonization period of *N. seriolae* (Teng et al., 2022, 2023).

Upon entry of *N. seriolae* into fish organs, local macrophages phagocytose most of the bacteria (Zhang et al., 2024). However, this immune process may not effectively eliminate the bacteria, allowing *N. seriolae* to grow and multiply within macrophages. Infected macrophages can be deprived of their ideal niche for intracellular bacteria through induced apoptosis or cell death (Wang et al., 2009). In this study, integration of proteomic and transcriptomic data revealed significant enrichment of key proteins in the ferroptosis pathway following *N. seriolae* infection. Ferroptosis is a type of regulated cell death that is distinct from apoptosis. Nonetheless, this mechanism effectively aids in eliminating infected cells, thereby impeding bacterial replication and disrupting their life cycle (Farhadi et al., 2023). Tissue sections displayed pronounced granulomas in the spleen and kidneys, clusters of macrophages at the lesions, and extensive apoptosis and necrosis within the granulomas. Additionally, our previous study indicated that granulomas in the liver of snakeheads formed later than those in the spleen and kidneys, developing gradually after approximately 6 dpi (Teng et al., 2023, 2024). While no granulomas were observed in the liver at 4 dpi, a significant number of apoptotic and necrotic hepatocytes were present. Thus, cell death triggered by the ferroptosis pathway could serve as a protective mechanism in fish to halt the dissemination of *N. seriolae*. Iron storage is crucial for protecting cells from oxidative stress caused by an abundance of redox-active free iron. Our analysis revealed a significant increase in both mRNA and protein expression of *FTH1*, *FTL*, and *TFR1* following infection with *N. seriolae*. We hypothesize that the upregulation of FTL and FTH1 aims to protect intracellular cells in the spleen of the snakehead from ferroptosis by enhancing iron storage. Moreover, ACSL3 can convert monounsaturated fatty acids into acyl coenzyme A, which then binds to membrane phospholipids, to protect cells from ferroptosis (Magtanong et al., 2019). This may result in decreased availability of external iron necessary for *N. seriolae* proliferation, which directly inhibits the intracellular survival of the bacterium.

In this study, significant enrichment of the complement and coagulation cascades pathway was observed at 96 h. Complement factor B (*Bf*) is a serum glycoprotein that undergoes cleavage into Ba and Bb fragments upon activation of alternative pathways in the complement system (Li and Sun, 2017). Research has demonstrated a significant upregulation of *Bf* expression in the kidney, spleen, and liver of tongue sole (*C. semilaevis*) after bacterial infection in a time-dependent manner. Moreover, recombinant Ba from tongue sole exhibits remarkable binding affinity to bacteria and effectively inhibits bacterial growth upon binding (Li and Sun, 2017; Holland and Lambris, 2002). Similarly, fish complement factor H (*CFH*) has been reported to bind to invading pathogenic bacteria (Holland and Lambris, 2002; Wei et al., 2022). In our study, we observed a 2.18-fold upregulation in the expression levels of *Bf* gene and a 1.81-fold upregulation in *CFH* gene expression, suggesting the potential involvement of these two proteins in binding *N. seriolae* in snakeheads after infection. Previous studies have shown that overexpression of *c1qb* in a cell model of silver pomfret partially inhibits the reproductive and invasive capacity of *N. seriolae* (Li et al., 2024; Du et al., 2019). Furthermore, the production of C1q by myeloid lineage cells has been shown to enhance CD8^+^ T cell function (Eddens et al., 2024). In our study, the expression of both protein subunits of C1q (i.e., C1qb and C1qc) was notably upregulated in the spleen. Proteomic data indicated elevated mRNA and protein levels of key immune genes within the complement and coagulation cascades pathway, implying that this pathway serves as an essential component of the immune response in northern snakehead against *N. seriolae* infection. These cascades collaborate to strengthen the host’s defense against pathogens, highlighting their significance in immune defense mechanisms.

Chemokines serve as crucial inflammatory mediators, orchestrating the recruitment of macrophages and other immune cells to sites of infection or injury during acute inflammation (Sokol and Luster, 2015). In this study, the chemokine signaling pathway in the immune system subcategory exhibited the highest number of DEGs in the Cp versus Np comparison groups. Infections caused by pathogenic bacteria often trigger the expression of various chemokines in fish tissues. Notably, most chemokines were observed to be downregulated in hosts infected by *N. seriolae* (Chen et al., 2018; Byadgi et al., 2016). In the proteomic data, we also observed suppression of two chemokines (CXCL13 and CCL4) and related proteins (DOCK2, WASP, RAP1b, and PIK3CD) in the infected spleen. DOCK2, initially identified in lymphocytes and macrophages, is known to play crucial roles in chemotaxis for neutrophils, lymphocytes, and plasmacytoid dendritic cells (Fukui et al., 2001). A study by Jing et al. (2019) revealed that the loss of DOCK2 led to reduced activation of WASP and accelerated degradation of WASP, consequently impeding early activation of B cells. Furthermore, DOCK2-deficient T cells exhibit reduced antigen-specific proliferation (Randall et al., 2024). Importantly, this study demonstrated that both the T cell receptor signaling pathway and the B cell receptor signaling pathway were inhibited following *N. seriolae* infection. This implies that *N. seriolae* could potentially evade elimination by diverse immune cells through the suppression of the chemokine signaling pathway as well as T and B cell activation.

Notably, a co-expression network analysis revealed a strong correlation between *CLEC4E* and *RAP1b* and *PIK3CD* in the chemokine signaling pathway. The protein expression of RAP1b and PIK3CD was inhibited following infection with *N. seriolae*. Both *RAP1b* and *PIK3CD* are essential for activating the chemokine signaling pathway and for early B cell development. Based on these findings, it is further hypothesized that these processes are inhibited *in vivo* after infection with *N. seriolae.* Moreover, *CLEC4E* showed correlations with *MASP2*, *C5*, *Bf*, *SERPINF2*, and *CFH* in the complement and coagulation cascades pathway. In this study, CLEC4E, identified as a key protein, exhibited a significant upregulation of 19.56-fold following infection with *N. seriolae*. Previous research has indicated a significant upregulation of *CLEC4E* in various tissues following infection of turbot (*Scophthalmus maximus*) with *V. anguillarum* and *E. tarda* (Zhu et al., 2021). Johansson et al. (2016) found that the CLEC4E homolog in rainbow trout induces expression in macrophages when stimulated with lipopolysaccharide. The homolog of CLEC4E in tongue sole also demonstrated a similar pattern (Jiang and Sun, 2017). Previous research has demonstrated the crucial role of CLEC4E in triggering autophagy through MYD88, which is essential for controlling *Mycobacterium tuberculosis* growth (Pahari et al., 2020). In addition, the Mincle encoded by *CLEC4E* recognizes the major mycobacterial virulence factor trehalose-6′,6-dimycolate, initiating the Syk–Card9 signaling pathway in macrophages to combat mycobacterial infections (Bowker et al., 2016). It is worth noting that *Nocardia*, phylogenetically linked to *Mycobacterium*, also induces lesions with similar structures (Shah et al., 2017). Hence, it is conceivable that the host may employ similar defense mechanisms against *N. seriolae* infection. However, further investigation is necessary to fully elucidate these mechanisms.

## 5 Conclusion

**Figure 10:**
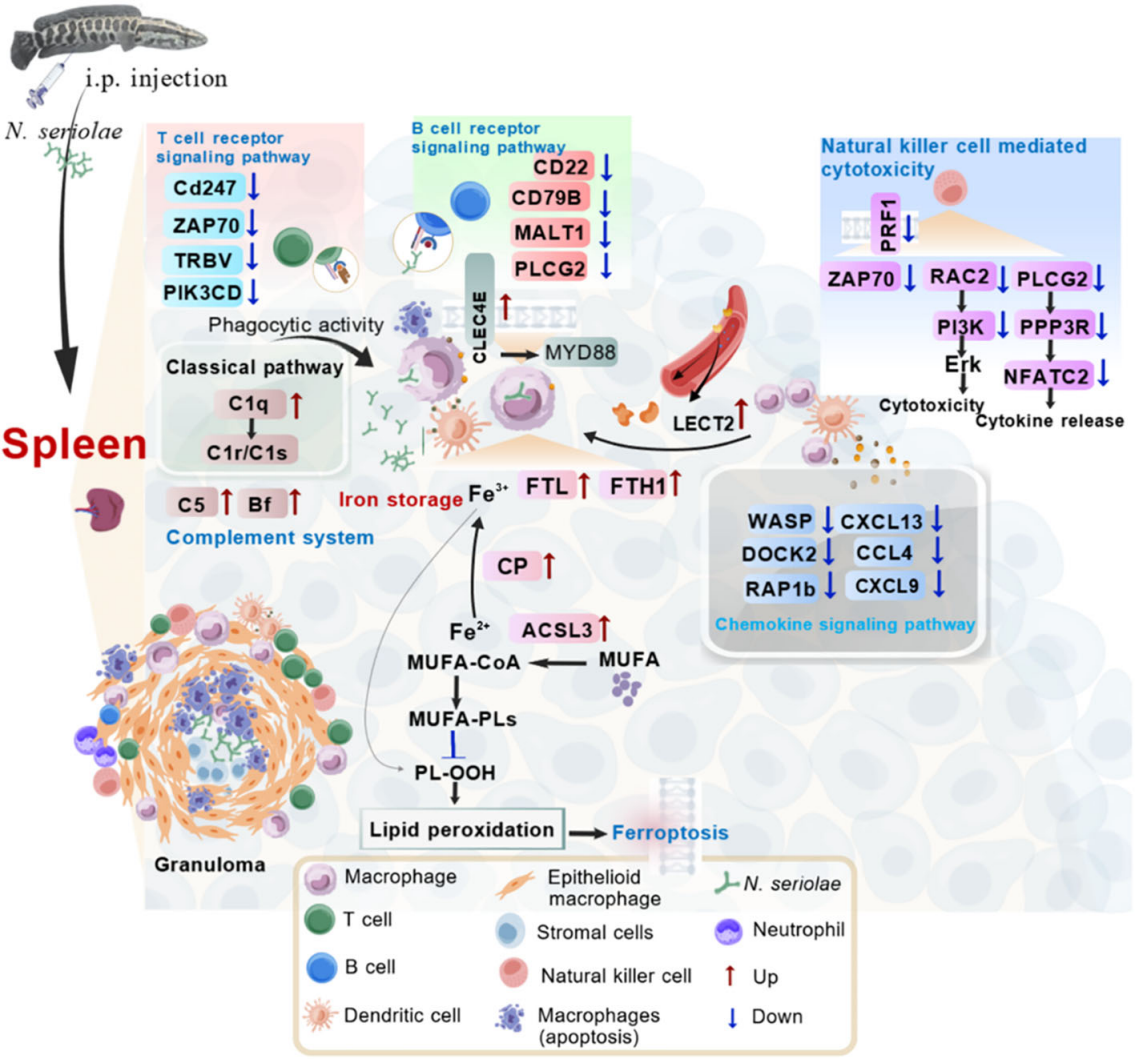
Hypothesized immune mechanisms of fish spleen against *N. seriolae* infection. The hypothesized portrait of activated immune pathways in the spleen of the snakehead was drawn based on the analysis of currently identified immune genes. During immune defense, a substantial number of genes with immunomodulatory functions were identified in the spleen, including innate immune molecules such as *CLEC4E*, *FTL*, *FTH1*, *C5*, *Bf*, *CFH*, *C1q*, and *LECT2*. These molecules are primarily involved in pathways associated with ferroptosis, the complement and coagulation cascades, the chemokine signaling pathway, and natural killer cell-mediated cytotoxicity. Moreover, genes associated with T/B cell responses, including *CD247*, *ZAP70*, *TRBV*, *CD22*, *CD79B*, and *MALT1*, demonstrated significantly reduced expression levels. Furthermore, a notable presence of apoptotic macrophages was observed within splenic granulomas. MUFA: monounsaturated fatty acids; PL-OOH: phospholipid peroxides. This graphic was created by BioGDP.com.

The present study used label-free protein quantitation to predict systemic responses in northern snakeheads following *N. seriolae* infection. The predicted pathways, including ferroptosis, complement and coagulation cascades, chemokine signaling pathway, tuberculosis, natural killer cell-mediated cytotoxicity pathways, offer valuable perspectives into the defense mechanisms of the spleen in northern snakeheads. Macrophages utilize these activated immune responses to eliminate the intracellular niche of the pathogen or to aggregate at bacterial colonization sites to restrict pathogen diffusion (**Figure 10**). The activation of these pivotal pathways in the early stages of infection appears to assist in suppressing *N. seriolae* proliferation and dissemination. Further studies are needed to address the functions of these DEPs in immune response and protection against *N. seriolae* infection. Such investigations are essential for effective disease control and maximizing aquaculture yield.

## Data Availability

The data presented in the study are deposited in the Proteome Xchange Consortium repository and the Short Read Archive of the NCBI repository, with accession numbers PXD041478 and SRP302800, respectively.
