# Supplementary material for: Integrated analysis of proteome and transcriptome revealed changes in multiple signaling pathways involved in immunity in the northern snakehead (Channa argus) during Nocardia seriolae infection
Source: Front Cell Infect Microbiol. 2024 Dec 9;14:1482901. doi: 10.3389/fcimb.2024.1482901 (PMC11663741; doi:10.3389/fcimb.2024.1482901)
Supplement: Supplementary file 1 [file Table1.docx]

**Table S1**

Primers in this research

| Genes | Forward primers (5’-3’) | | Reverse primers (5’-3’) | ID |
| --- | --- | --- | --- | --- |
| *CTSS* | | CCAGAATGAGGTGGAGGATG | GTCAGTTGGAGGACGGAGTG | Carg_Chr09G011070.1 |
| *ATP6V1A* | | GTATCCAGCGACCCCTAAAG | GTGATGTGACTGCCAACCCG | Carg_Chr22G003060.1 |
| *Prf1* | | AARGACAAAACATGTACCCT | TTTAGSTCTCCAATCCACCA | Carg_Chr08G011210.1 |
| *IRF4* | | CTTGTATGCTAAGCGTCTGTG | CCTGGTGTCTGGGTATATGTC | Carg_Chr16G001080.1 |
| *Stat3* | | ATCTGGCTATGGTGCTGTTC | TATGGCGTCTAATGTTGTGC | CF_GLEAN_10008827 |
| *ACSL1* | | CCACTCTACGACCTCGACAT | GAACCAAGAAACGGACCATT | CF_GLEAN_10005104 |
| *TFRC* | | TGTTCCTATCAGTTCAGCCTC | CCTTTTTACATCATCCCAGT | CF_GLEAN_10020177 |
| *krt18*  *nccrp1*  *β-actin* | | TTCTGCCCACATCTCCTTGAC  GCTATGGACCTGGGGTGAGAT  CACTGTGCCCATCTACGAG | CCCATACCACCACCTATTCCA  GCAAAGAGTGTGGGGAAGAAT  CCATCTCCTGCTCGAAGTC | CF_GLEAN_10006161  CF_GLEAN_10016680  Chen et al., 2018 |


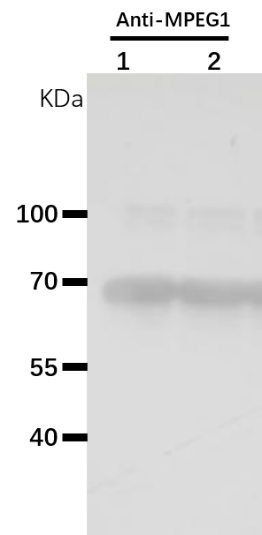


**Figure S1.** The specificity of the anti-MPEG1 antibody was detected by western blotting. Lane 1 and lane 2 represent spleen samples used as antigenic proteins at 24 and 96 hours after challenge with *N. seriolae*, respectively.


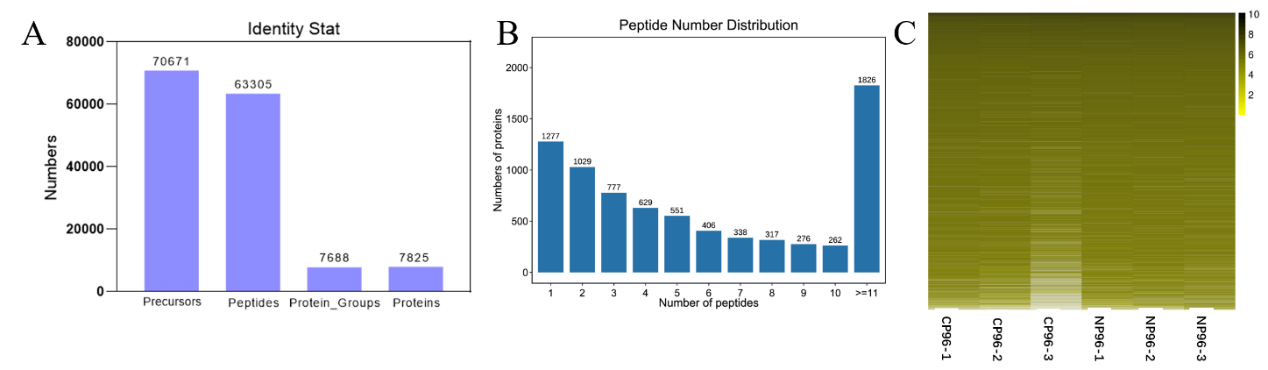


**Figure S2.** Overview of the global proteomic data in this study. (A) Statistical analysis for protein identification. (B) Basic statistics for peptide fragments used in the protein identification. (C) Heatmap of protein quantification for CP and NP group. Each column represents a single individual and each row a unique protein. The heatmap is displayed on a log10 scale, with deep yellow indicating higher expression levels, pale yellow indicating lower expression levels, and white indicating the absence of a protein in the sample.


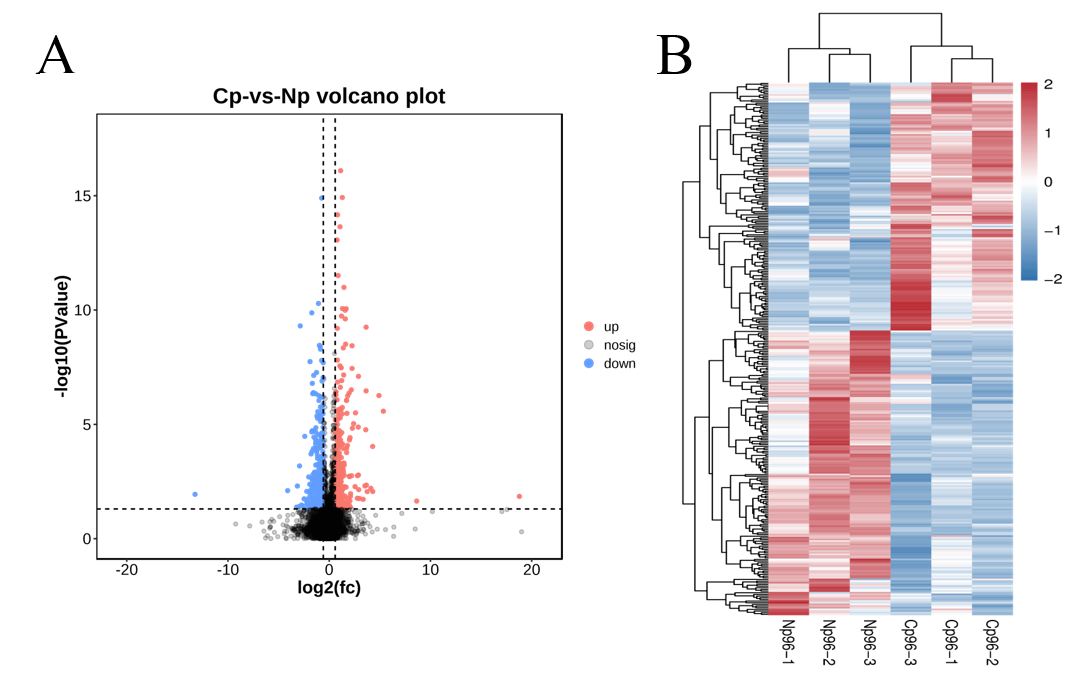


**Figure S3.** Differential protein expression between northern snakeheads infected with and without *N. seriolae*. (A) Volcano plot comparing the abundance of proteins in the NP and CP groups. Red dots represent significantly up-regulated proteins, blue dots represent significantly down-regulated proteins, and black dots represent proteins with no significant difference. (B) Hierarchical cluster analysis of differentially expressed proteins (DEPs) in the CP and NP groups. Each column represents a sample, and each row represents a protein. Red indicates high protein expression levels, while blue indicates low protein expression levels.


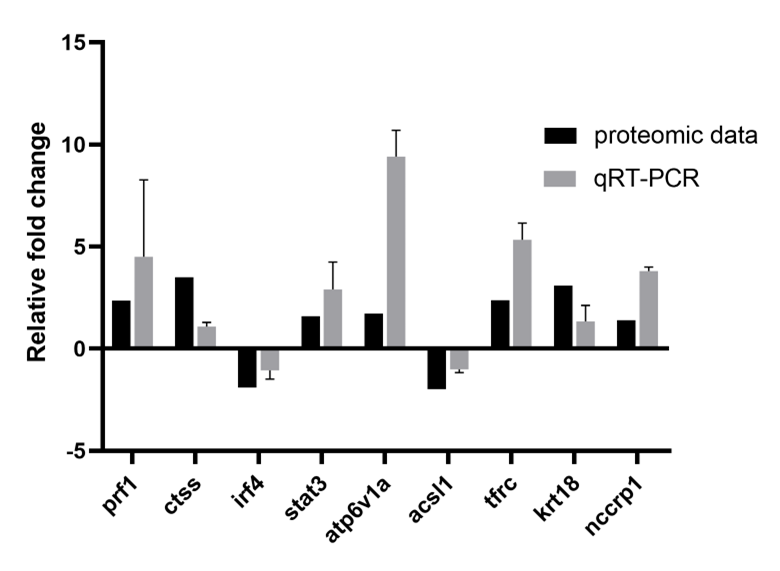


**Figure S4.** Comparison of the expression profiles of eight genes as determined by proteome and validated by qRT-PCR. Data represent the means for three independent experiments and error bars indicate SD.


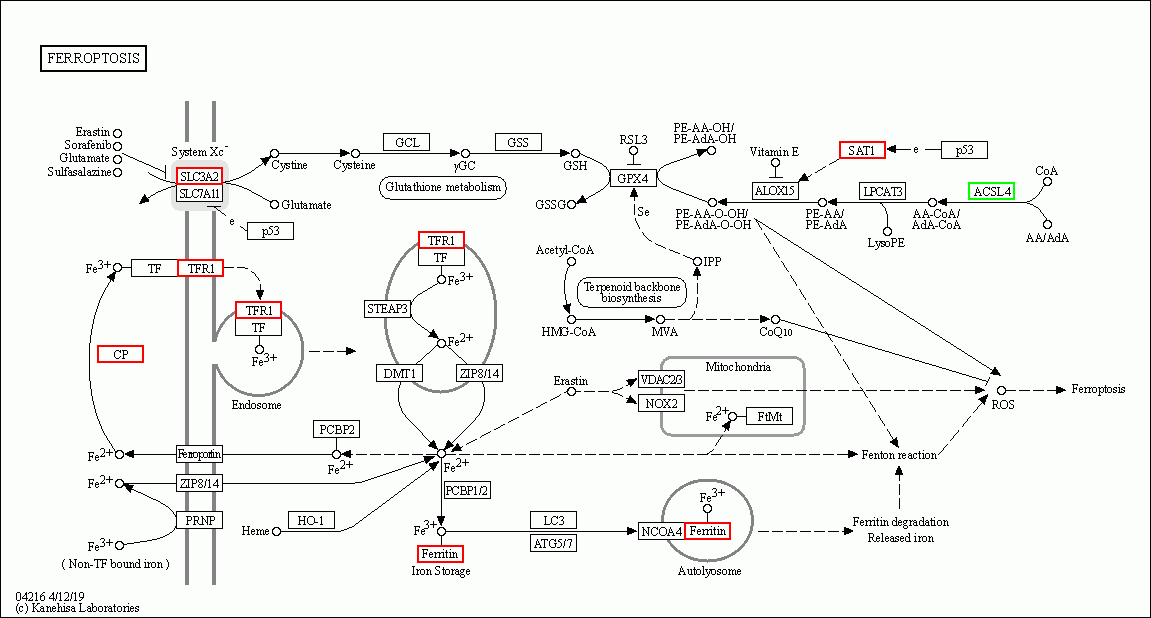


**Figure S5.** An overview of the Ferroptosis pathway in northern snakehead. The up-regulated genes were labelled with red, while the down-regulated genes were labelled with green. Undetected genes are shown by white coloring. The frames in both red and green indicated that these genes had more than one DEPs.

**References**

Chen, J., Li, Y., Wang, W., Xia, L., Wang, Z., Hou, S., et al. (2018). Transcriptome analysis of immune-related gene expression in hybrid snakehead (*Channa maculata* ♀ × *Channa argus* ♂) after challenge with *Nocardia seriolae*. *Fish Shellfish Immunol*. 81, 476-484. [doi](https://doi): 10.1016/j.fsi.2018.07.039
